# Editorial: Metabolic Abnormalities and Breast Cancer: Challenges From Bench to Bedside

**DOI:** 10.3389/fonc.2022.890810

**Published:** 2022-04-27

**Authors:** Zheng Wang, Pu Li, Mark Daniel Pegram, Xiaosong Chen

**Affiliations:** ^1^Department of General Surgery, Comprehensive Breast Health Center, Ruijin Hospital, Shanghai Jiao Tong University School of Medicine, Shanghai, China; ^2^Ruijin Hospital, Shanghai Jiao Tong University School of Medicine, Shanghai, China; ^3^Stanford Cancer Institute, Stanford University School of Medicine, Stanford, CA, United States

**Keywords:** breast cancer, metabolism abnormalities, metabolic reprogramming, tumor microenvironment, obesity

Breast cancer (BC) ranks as the first malignant disease and the second leading cause of death by cancer in women (1). Despite the development and progress in multi-disciplinary treatments, metastasis and drug resistance pose a huge impediment to further improving the clinical outcome of BC patients. Cancerous cells own the capacity to get nutrition in a tough environment in order to sustain the transformed state, build biomass, and promote proliferation and invasion (2). Thus, metabolic characteristics play an important role in the formation, invasion, and metastasis of breast cancer as well as the development of therapeutic resistance.

Literature reported that the tumor progression, treatment response, and clinical outcome would be affected by host metabolic abnormalities, including diabetes, obesity, and metabolic syndrome (3). Meanwhile, metabolic reprogramming is termed as another emerging hallmark of breast cancer. The distinct metabolic phenotypes of tumor cells, such as glycolysis and altered metabolism of carbohydrates, fat, and protein, could facilitate the tumorigenesis and evolution of breast cancer, which opens up a new scenario for overcoming disease progression (4). In addition, the metabolic pathway of BC cells alters with the tumor microenvironment, including fibroblasts, immune cells, and adipocytes, which ultimately lead to changes in cellular behavior (5). Thus, elucidating the molecular mechanisms, clinical implications, and potential targets involved in the above metabolism abnormalities can further reveal the biology of breast cancer and improve the prognosis of patients. Under this circumstance, the current Research Topic collected 17 scientific studies (11 original research articles, and six reviews) focused on metabolic abnormalities and breast cancer. Those researches narrate the latest progress and reviewed the recent advances in this field, from the basic, translational and clinical aspects.

In the review by Dong et al., metabolic syndrome could affect prevalence, treatment response, progression and survival of breast cancer. As for the initiation of breast cancer, Yan et al. studied the association between mammary tumorigenesis and metabolome in a novel mouse model and showed that MCP-1 derived from adipose could contribute to breast tumorigenesis. By performing a nationwide population-based cohort study, Seol et al. found that elevated GGT level could be a risk factor for breast cancer, especially in the obese post-menopausal group.

In terms of progression and metastasis, Wang et al. illustrated that Lactate Dehydrogenase-A (LDHA) mediated a loop between breast cancer stem cell plasticity and tumor-associated macrophage infiltration, which would a potential target for combating metastasis. Since both intrinsic and extrinsic factors contributed to metabolic reprogramming phenotypes, Wang et al. comprehensively reviewed the metabolic mechanisms underlying BC metastasis.

Several authors focused on the therapeutic response and resistance. Lu et al. found that the UCHL1, a deubiquitinating enzyme, could lead to chemoresistance by modulating free fatty acid synthesis. Wang et al. found that pyrotinib and adriamycin had synergistic effects on HER2-positive BC. Qiu et al. reviewed the newly published studies in the correlation between hyperglycemia and chemoresistance, as well as the hyperglycemic microenvironment and glucose metabolism. Li and Li. further summarized the recent studies about mitochondrial metabolism and therapeutic resistance in breast cancer. And Wang et al. made a review of the mechanisms by which salinomycin protected against breast cancer and discussed its future clinical applications. In additional, He et al. studied the lipid changes during endocrine therapy and found that tamoxifen would improve total cholesterol and low-density lipoprotein levels in premenopausal patients, and that aromatase inhibitors had no adverse effects on lipid profiles.

Furthermore, Qin et al. studied the relationship between hormone receptor status and prognosis between medullary breast carcinoma and atypical medullary carcinoma of the breast. And Tong et al. analyzed the correlation between 21-gene recurrence score (RS) and obesity, indicating RS varied among different obesity status.

Several articles studied the molecular mechanism beyond metabolic changes in breast cancer, including non-coding RNAs, RNA modification, and hypoxia. Du et al. and Ni et al. indicated that long non-coding RNAs MIR210HG and ADAMTS9-AS2 could modulate the metabolic reprogramming and progression of triple-negative breast cancer (TNBC). Sheng et al. further illustrated the latest progress in DNA N6-Methyladenine modification and drug resistance in TNBC. And Zhang et al. reviewed the role of hypoxia in breast cancer, and discussed the relationship between hypoxia and therapeutic response, as well as the clinical values of hypoxia biomarkers.

In conclusion, all these publications in the present Research Topic provide new insights into the role of metabolic abnormalities in the disease development, treatment response, and prognosis of breast cancer. We hope that the finding of the articles in this Research Topic would provide novel treatment strategies to improve survival of BC patients.

## Author Contributions

All authors contributed equally to this Editorial. All authors contributed to the article and approved the submitted version.

## Conflict of Interest

The authors declare that the research was conducted in the absence of any commercial or financial relationships that could be construed as a potential conflict of interest.

## Publisher’s Note

All claims expressed in this article are solely those of the authors and do not necessarily represent those of their affiliated organizations, or those of the publisher, the editors and the reviewers. Any product that may be evaluated in this article, or claim that may be made by its manufacturer, is not guaranteed or endorsed by the publisher.
